# The *Saturation*+ Approach to Behavior Change: Case Study of a Child Survival Radio Campaign in Burkina Faso

**DOI:** 10.9745/GHSP-D-15-00049

**Published:** 2015-11-03

**Authors:** Joanna Murray, Pieter Remes, Rita Ilboudo, Mireille Belem, Souleymane Salouka, Will Snell, Cathryn Wood, Matthew Lavoie, Laurent Deboise, Roy Head

**Affiliations:** ^a^​ Development Media International, London, UK; ^b^​ Development Media International, Ouagadougou, Burkina Faso

## Abstract

A 35-month cluster randomized controlled trial was conducted in Burkina Faso to test whether a radio campaign focused on child health, broadcast between March 2012 and January 2015, could reduce under-5 mortality. This paper describes the design and implementation of the mass media intervention in detail, including the *Saturation+* principles that underpinned the approach, the creative process, the lessons learned, and recommendations for implementing this intervention at scale. The *Saturation+* approach focuses on the 3 core principles of saturation (ensuring high exposure to campaign messages), science (basing campaign design on data and modeling), and stories (focusing the dramatic climax on the target behavior) to maximize the impact of behavior change campaigns. In Burkina Faso, creative partnerships with local radio stations helped us obtain free airtime in exchange for training and investing in alternative energy supplies to solve frequent energy problems faced by the stations. The campaign used both short spots and longer drama formats, but we consider the short spots as a higher priority to retain during scale-up, as they are more cost-effective than longer formats and have the potential to ensure higher exposure of the population to the messages. The implementation research synthesized in this paper is designed to enable the effective adoption and integration of evidence-based behavior change communication interventions into health care policy and practice.

## Abstract (precis)

This randomized radio campaign focused on the 3 principles of the *Saturation+* approach to behavior change: (1) saturation (high exposure to messages), (2) science (basing design on data and modeling), and (3) creative storytelling. Locally developed short spots and longer dramas targeted multiple child survival-related behaviors and were delivered entirely by local radio stations. Innovative partnerships with radio stations provided free airtime in return for training, equipment, and investment in solar power.

## INTRODUCTION

Between March 2012 and January 2015, Development Media International (DMI) implemented a 35-month mass media campaign in Burkina Faso and tested the impact of this intervention on child mortality through a cluster randomized controlled trial (RCT), funded by the Wellcome Trust and Planet Wheeler Foundation.^1^^,^^2^ The media intervention consisted of daily radio broadcasts (60-second spots and longer, interactive dramas) that targeted changes to multiple, key behaviors to improve child survival. We broadcast in 7 randomized geographic areas (clusters, which correspond to areas covered by local community FM radio stations) across Burkina Faso, with 7 additional clusters serving as controls. The RCT tested the impact of a mass media campaign alone; there was no supply-side intervention.

This trial is the largest, most rigorous evaluation of a mass media intervention in a low-income setting. The independent evaluation is being led by the London School of Hygiene and Tropical Medicine (LSHTM) in partnership with Centre Muraz in Burkina Faso. Household surveys were conducted at baseline, midline, and endline to measure family behaviors and at baseline and endline to measure under-5 child mortality. The midline survey methods and midterm behavioral results have been published in a companion article in *Global Health: Science and Practice*.^3^ Effects on behaviors and child mortality at endline will be published as results become available.

The aim of this paper is to describe the implementation of our mass media intervention in detail. First we document the *Saturation+* principles and theory of change underpinning our approach. Then we describe our implementation of the Burkina Faso campaign, its design, execution, our creative process, and how these components follow the *Saturation+* principles. Finally we discuss the lessons learned and our recommendations for implementing this intervention at scale. We believe this type of implementation science is crucial to enabling effective replication of evidence-based interventions. A lack of such information too often impedes the integration of research findings into health policy and practice.^4^

## THE *SATURATION+* APPROACH

The media intervention tested in the trial was designed and implemented based on DMI’s *Saturation+* approach, which builds on existing communication principles for behavior change. It was developed following many years of experience in designing, implementing, and evaluating media campaigns.^1^

The *Saturation+* approach is a set of core transferable principles, grouped under the 3 main categories of saturation, science, and stories (Box 1). It is not intended to be a standard, one-size-fits-all method, but rather an approach designed to maximize the impact of campaigns. If successful, the trial will not prove that *any* mass media campaign can reduce child mortality. Rather, it will provide evidence of the impact of the specific *Saturation+* approach that underpins the media intervention being tested.

The *Saturation*+ approach to behavior change focuses on the 3 principles of saturation, science, and stories.

**BOX 1. Core Principles Underlying the Saturation + Approach to Behavior Change**

Saturation

- Analyze media penetration data to reach the largest possible proportion of the target audience.
- Develop partnerships with market-leading stations.
- Devise radio/TV formats that can be produced at a rapid rate to enable frequent broadcasts.
- Broadcast messages in local languages, several times a day, for a sustained period (6–12 times per day for radio spots, at least 3 times per day for TV spots, at least once a day for other formats).

Science

- Use mathematical modeling to estimate the health impact of each message.
- Design calendar of messages that prioritizes highest-impact messages.
- Conduct formative research to understand target audience and barriers to behavior change.
- Summarize research in 1-page message briefs to inform the creative process.
- Pretest spots to ensure message clarity and acceptability.
- Monitor audience reaction to broadcasts.
- Conduct a robust quantitative evaluation to enable measurement and attribution of impact.

Stories

- Recruit talented local scriptwriters, using open competitions where appropriate.
- Build dramatic structures in which the emotional climax addresses the crucial barrier to behavior change.
- Use an editorial process that ensures quality control while allowing space for creativity.

## Saturation Theory

Intensity is key to any commercial advertising strategy, and yet it has been an underrated element of public health campaigning. Evidence suggests that achieving high exposure to messages is correlated with impact on behaviors.^5^ Although much of the existing evidence base comes from non-randomized evaluations (which may be affected by confounders), many of these studies make a strong case for the attribution of effects to their campaigns by showing that higher exposure is associated with incrementally higher impact on outcomes (dose-response effect).^6^ For example, the COMMIT media trial demonstrated correlation between exposure to the intervention “dose” and the reported effect “response” on smoking cessation.^7^

High exposure to behavior change messages is correlated with impact on behaviors.

A recent systematic review of the effectiveness of mass media interventions for child survival in low- and middle-income countries reported that achieving adequate exposure is a key component of success, with campaigns needing to “reach substantial proportions of the target audience with enough frequency to be recalled.”^8^ Nearly one-third (31%) of the studies included in the review had achieved exposure of 61% to 100%,^8^ which has been shown to be a strong predictor of campaign success.^9^ Another review of the impact of media campaigns on health behaviors proposed that investment in longer, better-funded campaigns is required to achieve frequent and widespread population exposure to messages, especially for habitual behaviors.^10^ Our own experience also indicates a link between the frequency of messaging and impact on behavior change. A particularly successful campaign targeted hand washing in Ethiopia, with messages broadcast up to 14 times per day for 3 years.^11^^,^^12^ Further analysis of data from both published studies (by Roy Head in consultation with the studies’ first author, Tansy Edwards) suggests significant reductions in observed dirty hands (decreasing from 74% to 26%) and a 20% reduction in the prevalence of trachoma in areas receiving radio messages alone without the use of antibiotics.

So *how* does exposure lead to behavior change? There are several theories about the mechanisms or pathways by which high exposure drives behavior change, summarized by Bob Hornik, including^5^:

- **Learning.** People listen to the radio at different times each day and vary in their susceptibility or inclination to respond to a message. The more times a message is repeated, the more *opportunities* there are for people to hear and learn from the message when they are receptive to it.
- **Priming.** Repeated exposure to a message affects its pertinence, so a stronger weight is attributed to the message when deciding whether to adopt the behavior.^13^
- **Creating social norms.** Repeated exposure to messages can create social expectations about behaviors. Such social norm pressures may persuade people to adopt behaviors.^14^
- **Diffusion effect.** As more people are exposed to messages, more people will discuss these messages within their wider social networks, including people who have not seen or heard the media campaign.^15^
- **Indirect impact on policy.** High exposure may alert policy makers to issues that are of public concern and thereby result in legislation or the implementation of policies that promote behavior change.

**FIGURE 1.**
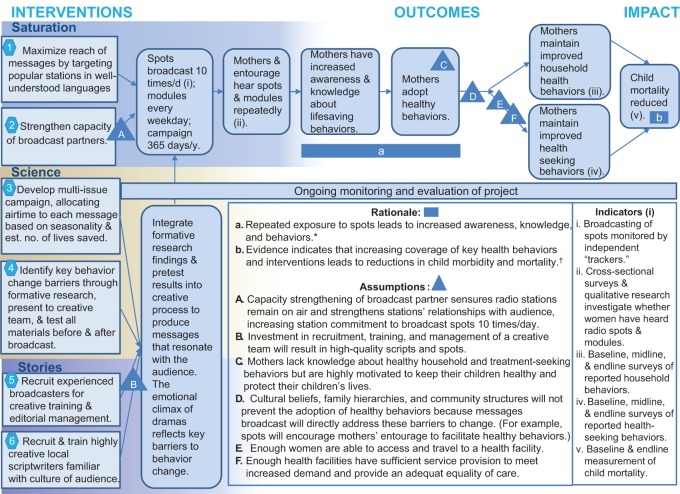
Theory of Change for the *Saturation+* (Saturation, Science, and Stories) Mass Media Campaign in Burkina Faso ^*^ Rationale articulated in Hornik, 2002.^5^ † Sources: Lancet Child Survival Series 2003; Steinglass R, Cherian T, Vandelaer J, Klemm RD, Sequeira J. Development and use of the Lives Saved Tool (LiST): a model to estimate the impact of scaling up proven interventions on maternal, neonatal and child mortality. Int J Epid. 2011;40(2);519-520; Fox MJ, Martorell R, van den Broek N, Walker N. Technical inputs, enhancements and applications of the Lives Saved Tool (LiST). BMC Public Health. 2011;11 Suppl 3; and Lassi ZS, Salam RA, Das JK, Bhutta ZA. Essential interventions for maternal, newborn and child health: background and methodology. Reprod Health. 2014;11 Suppl 1:S1.

In the particular case of child mortality, there is another mechanism at work. The primary audience is mothers, and the motivation of mothers to protect their children is one of the strongest instincts in nature. While we know that knowledge alone is an insufficient instrument in, say, antismoking campaigns in which motivations are complex, it is safe to assume that virtually all mothers are highly motivated to protect their child with the proper knowledge of how to do so. Ensuring—through repeated broadcasts—that they are *aware* of danger signs and have the *knowledge* to protect their child will propel them further along the path to behavior change than less primal, instinct-driven behaviors. The broad theory of change or causal pathway by which we hypothesized that the Burkina Faso campaign would achieve impact is illustrated in Figure 1.

Bob Hornik, when summarizing his 2002 analysis of whether public health communication can change behaviors, suggested^16^:

Most of the innovative work in public health has focused on the problem of developing high quality messages. … This has been a good thing. At the same time, there has been less attention to the problem of exposure to those messages. … And that may be a crucial failing.

Irrespective of the specific pathways through which high exposure drives behavior change, both evidence and theory (not to mention a century of experience from the advertising industry) suggest that high frequency of messaging is a crucial component of successful health communication campaigns. We therefore consider it a crucial component of the *Saturation+* approach.

## IMPLEMENTATION OF THE BURKINA FASO CAMPAIGN

In this section we describe in detail the design and execution of the media intervention tested in the RCT in Burkina Faso, within the *Saturation+* framework. We have also produced an open-access *Saturation+* handbook (http://www.developmentmedia.net/saturation-handbook). This tool is designed for use by other organizations that are delivering mass media behavior change campaigns. While our mass media campaigns focus on child survival and the handbook was written with this subject in mind, the principles described are applicable to campaigns addressing other health and non-health subjects.

### Saturation

The broadcasting environment in Burkina Faso is unusual. Most people listen to local FM radio, rather than to the national station, because most output on the national station is in French (spoken by fewer than 1 in 5 rural Burkinabés) while the output of local FM stations is in the local languages. This environment makes it uniquely suitable for a randomized controlled trial.

Most people in Burkina Faso listen to local FM radio rather than to the national station, providing a uniquely suitable environment for an RCT.

Radio penetration in Burkina Faso is also high. The 2010 Demographic and Health Survey (DHS) reported that 68.3% of households owned a radio (65.7% in rural areas) while only 16.2% of households owned a television (5.8% in rural areas). In preparation for the trial, DMI and Centre Muraz conducted a media survey in 2011 to measure radio penetration in 19 rural areas and to identify the stations with the greatest number of female listeners. We found that 75% of women surveyed listened to the radio at least once a week. It was clear that radio is the only form of mass media currently capable of reaching our primary target audience (mothers of children under 5 years, pregnant women, and mothers-to-be) as well as the secondary audience (people who can directly influence the primary audience, in this case, the husbands and the mothers-in-law).

Saturation broadcasting can be achieved by paying the market-leading radio and television stations for airtime, but our experience is that it is much easier and cheaper to achieve saturation broadcasting if the broadcast industry is involved as a core partner. Unlike advertising agencies or governments, broadcasters have production capacity and airtime, the two vital ingredients of a media campaign. In the majority of our campaigns, we have negotiated free airtime in exchange for on-the-job training (working with each team to produce live evening programs) and for production expenses; the opportunity to secure better skills and advantage within a competitive media environment is usually enough incentive for broadcasters to partner with us. Our experience elsewhere has been that both private and public media organizations have been very willing partners. This was certainly the case in Burkina Faso.

The simplest way to achieve high intensity is to use short (e.g., 60-second) spots, as exemplified by the advertising industry. This format allows frequent daily broadcasts, across all peak listening times. It also allows us to produce precise health messages across a diversity of languages. The spots use emotion, humor, and dramatic techniques such as suspense to persuade our target audience to change behaviors. In our Burkina Faso campaign, we broadcast a new spot every week, played at least 10 times per day, over 35 months (from March 2012 to January 2015).

Short radio spots allow for frequent daily broadcasts across peak listening times.

Exposure through multiple channels is also associated with greater impact.^5^ In countries with sufficient television penetration and/or higher literacy levels, other communication channels would strengthen the impact of a campaign. Due to low television coverage in rural Burkina Faso, our campaign was broadcast on radio alone. However, we used multiple formats to deliver our messages on the radio.

To be effective, longer formats also need to reach audiences frequently. Throughout the campaign, we broadcast 2-hour interactive programs, 5 nights per week, on each of our 7 partner radio stations (one in each intervention zone), representing a total of 70 hours per week of live radio in 6 different languages. We therefore needed to devise a format that could deliver health messages, was cheap, could be broadcast daily, could be produced “live” (which costs a fraction of pre-produced radio) and yet could be controlled centrally. Producing a pre-recorded soap opera, for example, in 6 different languages would be expensive and logistically very difficult. So we created a system of self-contained drama “modules” that were written in French in the capital city, sent on USB keys through local transport companies to our partner radio stations, and improvised live by local actors on location in their own language within their 2-hour shows. These were followed by phone-ins to allow listeners to comment on the issues raised. The modules were around 10 minutes each, with the remainder of the 2-hour program taken up by news, music, and discussion. They were aired in the evening, which our research found to be the peak time for radio listening among our target audience. This program format works well in a fragmented media environment, which is becoming the norm in most developing countries. These longer dramas and interactive shows can add value by creating role models, demonstrating life skills, or allowing on-air dialogue with the public. They also help us to build relationships with partner stations and can thereby help to ensure that our spots are broadcast at the intensity required.

In Burkina Faso we used a system of broadcast monitoring to verify whether stations had played the spots as they had agreed, at the frequency required, and to allow swift remedial action if they had not (in our experience this was usually due to inefficiency or genuine technical problems). We hired two independent monitors or “trackers” in each radio station’s coverage area to record when our materials were broadcast, and we collected their results by telephone weekly. The radio stations were not aware of the identity of the monitors, nor were the two monitors aware of each other. Radio station compliance during the RCT was extremely high, with stations reliably broadcasting an average of at least 10 spots every day. Where feasible, radio programming software can also be used to monitor and verify spot broadcasting. Data on our broadcasting intensity, by health topic and by format, are presented in the companion midline results paper.^3^

### Science

Data and modelling should underpin the key aspects of campaign design. We use data in our campaigns in several ways. First, we use data to quantify the geographic coverage, audience size, and market share of different media channels in different parts of the country, at different times of day, and with different demographic groups. It is often difficult to obtain these data, but it is essential if resources are to be allocated correctly. In Burkina Faso, for example, we conducted a customized survey to estimate the market share and audience penetration of each radio station.

Data and modeling should underpin key aspects of campaign design.

We also use data to prioritize which messages can save the most lives per dollar spent. For this analysis, we use data on the mortality risk of different diseases, their susceptibility to behavior change, current levels of behavioral compliance, and the availability of key medical services. Our modeling work brings these data together,^1^ allowing us to predict the impact on mortality of each target behavior (using the Lives Saved Tool [LiST]^17^) and to weight campaign messages so that those predicted to save the most lives can be broadcast most frequently (Table).

**TABLE:** Lives Saved Model Predictions Used to Weight Messaging When Designing the Burkina Faso Radio Campaign

Theme of Messages	No. of Lives Saved According to Modeling^a^	Approximate No. of Months of Broadcasting
ORT	3000+	6
Malaria	3000+	7
Breastfeeding	2000+	5
WASH	2000+	4
Pneumonia	1000–1500	4
Extra care for low birth weight infants	1000–1500	3
Complementary feeding	500–1000	3
Maternal health	500–1000	3

Abbreviations: ORT, oral rehydration therapy; WASH, water, sanitation, and hygiene.

a Number of lives predicted to be saved if the campaign were implemented at national scale (not in the RCT intervention zones alone). These were the original model predictions at the start of the campaign. Message weightings were altered further during the course of the campaign.

**FIGURE 2.**
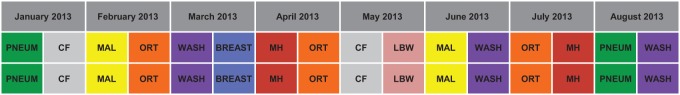
Weekly Radio Spots Messaging Calendar for the Burkina Faso Campaign, January–August 2013 Key: BREAST, early and exclusive breastfeeding; CF, complementary feeding; LBW, care of low birth weight infants; MAL, malaria prevention and treatment seeking; MH, maternal health: antenatal care attendance and delivery in a health facility; ORT, treatment seeking for diarrhea and use of oral rehydration solution and increased liquids; PNEUM, seeking treatment for pneumonia symptoms; WASH, water, sanitation, and hygiene.

For our campaign in Burkina Faso, where our primary aim was to reduce child mortality, we developed a message calendar based on the predicted impact of each behavioral message on under-5 lives saved. Our calendar also took into account seasonality, so that, for example, messages on seeking treatment for malaria were broadcast more frequently during the months when malaria transmission is typically highest. Each week of the year was assigned a message theme for spots; the theme of the longer modules changed daily but followed the same weighting as the spots (Figure 2).

The independent evaluation of our campaign included a baseline and a midline (as well as an endline) behavioral survey of 5,000 mothers of a child less than 5 years old, after 20 months of broadcasting. Using the midline results, we revised our message calendar to maximize the impact of the remaining months of the intervention. The revised message weightings were calculated by taking into account several factors, including the impact the campaign had already had on each behavior (from baseline to midline), the broadcast dose for each message (from baseline to midline), and the predicted impact of each behavior for the remainder of the campaign (from midline to endline, modeled using LiST). We suggest that impact data collected during a campaign (when available) should be used to adjust message weighting to optimize impact.

Message quality is also crucial, and qualitative research is a key element to ensuring this quality. Qualitative research includes formative research (to identify barriers to behavior change), pretesting of radio spots (to judge comprehension and appeal), and feedback research (to find out whether people have heard and understood the messages and what the remaining obstacles to behavior change are). The key, as argued below, is to link findings from such qualitative research as tightly as possible to the creative process.

We employed a team of in-house qualitative researchers, who conducted formative research at the start of our campaign. The research consisted of semi-structured individual interviews and focus group discussions with mothers and fathers of young children and influential members of their entourage (spouses, grandparents, co-wives), as well as individual interviews with key informants such as religious leaders, district medical chiefs, health center staff, midwives, and community health workers. For each health behavior, we synthesized this research into a 1-page message brief that presented the key behaviors to promote including:

- Contextual information about the behavior, including Ministry of Health policy and guidelines, and information drawn from guidance from the United Nations Children’s Fund (UNICEF) and the World Health Organization (WHO)
- Analysis of key decision makers within the target audience for the specified behavior
- Context-specific barriers to behavior change
- Context-specific factors facilitating behavior change

An example message brief is provided in Box 2. We have found that reducing formative research down to a 1-page message brief for each target behavior is a critically important step in our creative process as it makes the research more accessible to the script writing team and helps to bridge the gap between research and creativity.

**BOX 2. Example of a One-Page Message Brief on Exclusive Breastfeeding From the Burkina Faso Radio Campaign**

Breastfeed exclusively for the first six months of life

Behavior to promote

All mothers should breastfeed their babies exclusively for the first six months of life. The secret to having enough breast milk is to exclusively breastfeed. If you do not have enough milk for your baby, breastfeed more frequently and you will produce more milk. If you add other drinks/concoctions or food, including water, to your baby's diet this will reduce your milk production.

Reasons

Breast milk production is dependent upon frequent breastfeeding. Interrupting breastfeeding by giving other liquids to a baby will decrease production of breast milk. Breast milk is the best and only food and the best and only drink that an infant needs for the first six months of life, even in hot and dry climates. Through breast milk, the baby receives defenses against diseases such as diarrhea and respiratory infections. Adding other foods or liquids can affect the health of a baby during the first six months of life, because these liquids or foods can be contaminated, which may cause diarrhea.

Barriers to behavior change

Sometimes mothers who breastfeed their babies are concerned when the baby wants to nurse more often than usual. They can attach this behavior to a lack of breast milk. A baby may want to nurse more often for several reasons: perhaps a phase of intense growth is happening; the baby might just be more hungry/thirsty; during an episode of illness babies may nurse more often; babies also may nurse more often when teething; or the baby may be in need of more comfort. A mother may interpret the cries of a baby as signs of hunger, which is often the case, but a baby may also cry because he is tired, or because he wants to burp or pass wind.

Mothers should understand that they can produce enough milk by breastfeeding more frequently. If they believe that their baby needs other liquids or supplementary foods, and they give these to their baby, they may reduce their milk production because if the baby breastfeeds less frequently, the mother will produce less milk. Other liquids/brews or foods do not help the baby, nor the mother, because these will increase the baby's risk of illness and the mother will then have to spend even more time taking care of a sick baby.

Many people think that exclusive breastfeeding is not possible because they believe that breast milk alone is not a sufficient source of nutrition or water for babies. Because of this they add other food supplements or water to the baby's diet.Water is given because they fear that a baby will “dry up” in Burkina Faso's extreme heat. People compare breast milk to solid foods that adults eat and believe that a baby will be more thirsty because of the fat content of breast milk. Breast milk is not considered a source of water for the baby.

Decision and influencers

Mothers-in-law or aunts accompany expectant mothers before and after delivery. They have a strong influence on exclusive breastfeeding as they will advise mothers to add water, herbal potions, or other foods during the first six months. Like many others they also believe that it is beneficial to give babies “welcome water” and/or other liquids that stimulate the newborn's appetite. These practices exist in most of Burkina Faso's ethnic groups. The mother's entourage can help by not insisting on the mother giving additional liquids or foods to a crying baby, but by giving the mother time to nurse until the baby is satisfied. Also the entourage has a great responsibility to ensure that pregnant women and mothers are well nourished.

Factors contributing to behavior change

Women attach great importance to breastfeeding. Children under six months are usually in close physical proximity to their mother (on their back) and thus breastfeeding is readily accessible and available. So, mothers can feed their baby when they need to and until the baby is done.

Water is the main component of breast milk (88%); it is particularly hydrating and quenches thirst. The other components (12%) are: carbohydrates, lipids, proteins, and micronutrients. At the start of feeding, breast milk contains a lot of water and minerals to hydrate. In the middle of a feed, proteins and lipids increase in quantity. At the end of each feed, fat is more concentrated in the milk and gives the baby a feeling of satiety. This signals the end of feeding for the baby. That is why it is necessary to breastfeed the baby for long enough at each breast.

Infants younger than six months are usually in close proximity to their mother. The infants are carried on their mothers' backs and thus breast milk is immediately accessible and available. Many mothers place great emphasis on breastfeeding and breastfeed their babies up to two years of age. Most children under three years of age are breastfed.

Our team of scriptwriters drew on these message briefs to create dozens of scripts. The best of these went through a validation process involving creative staff in both Burkina Faso and London before being produced. We pretested the spots (for clarity, popularity, and understanding) in multiple languages using focus groups before selecting the spots and distributing them for broadcast. Pretesting is essential for ensuring messages are well received by the target audience. For example, we pretested a spot in which a character impersonating a diarrhea germ had a discussion with a baby. Our target audience could not grasp the concept of bacteria/germs nor of young babies talking, so they did not understand this spot. Although they seemed to comprehend the health message within the spot well, it was rejected after the pretest because the confusion around the story would detract from the take-home behavior. In contrast, we pretested a spot that featured a beneficial “genie” telling a mother and grandmother who are about to press and discard the mother’s colostrum that this first milk is full of nutrition and protects the newborn against illness. This spot was accepted at pretesting, as it was a better fit to the Burkinabé context, in which people are very familiar with stories of genies.

Our qualitative researchers also conducted post-broadcast feedback research using focus groups to provide an understanding of audience reactions to our messages and to find out whether and why people who hear our messages have changed their behaviors (or not). After each trip, our researchers fed back their findings to the creative team, forming a continual feedback loop. We used the information gathered through pretesting and feedback research to continually refine our message briefs and to tailor our messages to target existing barriers to behavior change.

Our qualitative research team also helped us to monitor the availability of commodities to ensure we were generating demand that was met by sufficient supply. Throughout the RCT, we maintained strong links with the Ministry of Health, WHO, UNICEF, and other organizations working in Burkina Faso to help us track supply-side initiatives and to ensure that our messages were consistent with government policies. Our research team was able to provide insight into the availability and quality of services available on the ground through their visits to health centers and by liaising with district medical officers. This was particularly important for the effectiveness of our treatment-seeking messages, which were service-dependent. Although our program comprised a demand-side intervention only, had we found there were significant, frequent stock-outs of the essential medicines required to treat serious childhood illnesses, we might have needed to reconsider our approach to messages promoting service-dependent behaviors. Supply-side constraints will need to be taken into consideration when interpreting the generalizability of this RCT’s findings to other settings. We have taken supply-side availability into account in our scientific model, using the LiST tool to predict the impact of mass media campaigns on child morality in other settings.^1^

An independent economic evaluation of the trial (led by LSHTM) will estimate the cost-effectiveness of our intervention and will provide further insight into the costs of implementation, household costs from increased health service use, and health system costs associated with increased care seeking.

As mentioned previously, details of the independent evaluation of this intervention on child mortality will be published separately. In general, since randomization of media interventions is rarely possible, we advocate the use of time-series or other quasi-experimental study designs to evaluate media campaigns and enable attribution of impact. It is important when designing an evaluation to carefully consider which outcomes will be measured, using health outcomes where feasible, and measuring knowledge, attitudes, and behavioral outcomes as indicators along the causal pathway of behavioral change.

### Stories

Stories have resonated with human beings for thousands of years. We are drawn to drama in ways in which we are not drawn to data or facts. Stories allow us to identify emotionally with characters, and emotions—such as fear, status, and guilt—are powerful determinants of behavior.^18^ But how do stories work?

We are drawn to drama and stories in ways in which we are not drawn to data or facts.

Creativity is often the “black box” in theoretical discussions: it is difficult to define or measure. Nevertheless it is possible to use systems to understand and then enhance the creative process. Understanding the structure of stories is important.

Virtually every Hollywood film conforms to a 3-act structure: Act I in which characters are given goals; Act II in which obstacles are thrown in front of the characters; and Act III in which the characters either change their goals or overcome the obstacles. This structure mimics life itself for most of us and also mimics the process of behavior change. The emotional turning point of most stories is at the end of Act II, the moment of decision for the main character when s/he must choose between competing emotions. Formative research, when conducted with this in mind, can therefore go further than simply identifying obstacles to behavior change (e.g., cost, inconvenience). It can identify the most important emotion (e.g., fear or guilt) that prevents people from complying and the most important emotion (e.g., love) that motivates people to comply. The conflict between those two emotions can then be made the centerpiece of a story’s dramatic climax at the end of Act II.

Choices about how we behave are often made in response to deep underlying human impulses to be accepted by others, to fit in with our peers, and to imitate people we respect. In Burkina Faso, both our spots and our longer-format programs were based on engaging stories and characters that reached out to the audience, helping them to feel empathy for the characters and their situations. Our stories aimed to move audiences to examine the health choices made in their own lives. Once people are thinking about making changes, the stories then provide concrete ideas about how to do that. Box 3 provides an example spot on the theme of exclusive breastfeeding.

**BOX 3. An Example of a Short Spot About Exclusive Breastfeeding From the Burkina Faso Radio Campaign**

«Saved by his Tears»

[sounds of baby crying each time his grandmother speaks, and laughing each time his mother speaks]

MOTHER-IN-LAW: [plaintive] Your baby is four months old now. His lips are dry. It’s hot. Let me give him some water. [The cries of the baby get louder.] You see he’s crying!

DAUGHTER-IN-LAW: Mother-in-law, I’m going to breastfeed him. There is enough water in my milk to quench his thirst; it also has all the nutrients he needs to grow strong until he’s six months. [baby’s laughter] Other liquids could harm his health; they could put germs in his belly.

MOTHER-IN-LAW: No decoctions or water until he’s six months old! Impossible! I’m going to give him some water. [louder crying from baby] But y it’s strange how he’s crying … !

DAUGHTER-IN-LAW: Mother-in-law, compare my baby to Fanta’s baby who is always sick. Fanta gives her baby other liquids and that makes him ill. Let my child drink my breast milk and you’ll see he’ll never be thirsty. [sounds of baby suckling followed by baby’s laughter]

MOTHER-IN-LAW: [embarrassed] You’re right because your baby is healthy and growing up strong. I’m going to suggest to Fanta she does the same as you. That’s what your baby is trying to tell me with his laughter. [more laughter from baby]

Stories for public health programming must be driven by research, requiring the creative and research teams to work in close harmony—often a formidable challenge given that creative writers typically rely on their own judgment and imagination to create stories. This practical challenge is a microcosm of the wider challenge of bringing science and mass media together. One practical tool to enable collaboration is to develop succinct, 1-page message briefs that summarize the formative research, forming the foundation for the scriptwriters’ work. Another method is to send scriptwriters to help lead focus groups in the field, while also involving qualitative researchers in the radio production process. In Burkina Faso, we found that field visits motivated scriptwriters, provided new inspiration and ideas, and gave them valuable insight into the realities of rural Burkinabé life.

To maximize creativity and for quality assurance, we use multilevel systems of editorial control whereby, for example, 30 ideas per month are reduced to 6 for pretesting and to 4 for production. It is important that after pretesting, as well as after broadcast, qualitative research is fed back to scriptwriters. This feedback loop ensures an evolving creative process that continually responds to the target audience.

Grassroots recruitment of local scriptwriters is essential to develop a creative team that understands the language, context, and cultures of the target audience. Rather than try to outbid other NGOs for the existing talent pool, in Burkina Faso we advertised in university campuses, bars, and public meeting places and asked applicants to write and submit a story. This approach resulted in 600+ applications and, in our experience, yielded higher caliber staff than working through the existing NGO or media industries. We reviewed more than 600 scripts, interviewed 80 people, and hired 13 as scriptwriters. The hired scriptwriters had a diverse range of previous employment experience, ranging from teachers to a security guard, and originated from 8 regions of the country. Once hired, the team initially received training from experienced creative producers, and skills development was then sustained through weekly creative workshops.

Recruiting local scriptwriters is essential to develop a creative team that understands the language, context, and culture of the target audience.

## LESSONS LEARNED AND RECOMMENDATIONS FOR SCALE-UP

A crucial but too often neglected task for any implementing organization is describing in detail the many lessons learned during the trial of an intervention and explaining how these can be addressed to enable scale-up. There has been a tendency for public health research to overlook the importance of reporting in detail exactly *how* and *under which circumstances* interventions work.^19^ Testing the efficacy of an intervention in controlled trial conditions does not provide policy makers with sufficient information to guide their investments under the complexities of real-world conditions. Here, we describe the key lessons learned during our campaign in Burkina Faso, along with suggested adaptations and recommendations for rolling it out at a national scale.

Cash payments for airtime are not necessarily the most useful incentives to develop partnerships with radio and television stations. Likewise, providing training and capacity building in exchange for airtime payments may not be feasible in some settings. We have found in Burkina Faso that solving the frequent energy problems that affect community radio stations—frequent power cuts, broken generators, and rising costs of energy supplies—by installing solar power is a more powerful incentive to ensure that stations remain committed to a campaign. Logistically it would not be possible to install solar power immediately in every community radio station involved in national scale-up. It would, however, be feasible to commit to installing solar power in all stations over perhaps the first 12 months of a national program. While this approach imposes a large upfront cost during the early stages of a campaign, it actually offers significant efficiency savings over paying for airtime. We believe that this would apply to those countries across Africa that have primarily locally based radio markets and where similar difficulties with energy supplies hamper the implementation of high-frequency saturation broadcasting campaigns.

The primary purpose of our campaign was to reduce child mortality, so all operational decisions were designed to maximize our impact on that outcome rather than specifically targeted at building capacity. Despite capacity building not being our main objective, we built the capacity of radio station partners in several ways, through the provision of equipment, staff training, and technical support. Key lessons learned were the importance of maintaining close relationships with station managers and of regular site visits. This was crucial in ensuring that we were made aware of staff changes and power or equipment problems as soon as they occurred, thus preempting the danger of a station going off air.

Another tool to motivate media partners is providing them with impact data specific to their audience. Being able to feed back information to radio stations, showing them how their efforts have improved health outcomes in their area, is a powerful motivator. Furthermore, we have provided basic training in how this information can be used by the stations themselves to demonstrate their effect and sell their airtime to others, in order to build capacity and thrive in a competitive media market.

Taking a community radio intervention to scale in multilingual countries will inevitably require the translation and broadcasting of multiple messages. In Burkina Faso, for example, national coverage can be achieved by increasing our number of community radio station partners from 7 in the RCT to 29, taking the number of languages required from 6 to 12. Given this level of linguistic complexity, it is highly unlikely that qualitative research and pretesting of spots can be carried out in all languages. Despite this, we emphasize the importance of conducting formative and feedback research in as many languages and as many regions as is feasible. Wherever possible, we advocate training scriptwriters who speak local languages to assist the qualitative research team with pretesting and feedback research.

Financial constraints may prohibit the possibility of maintaining both spots and interactive program formats when scaling-up this intervention. The constituents of a *Saturation+* media campaign will vary depending on available funding and a country’s media landscape. What we have tested in Burkina Faso was specifically designed and adapted to the media context there. Results from the midline quantitative evaluation published in the companion paper showed some suggestions of a positive correlation between exposure to the radio spots and behavioral outcomes (each additional week of broadcasting led to approximately a 1% additional behavior change). There was no evidence of this dose-response relationship with exposure to longer-format programs. Our qualitative research also found that although people reported learning from both program formats, they tended to express a preference for spots, as they are easy to understand, are concise, and are frequently heard, and they depict captivating stories. Additional studies specifically comparing media formats would be required to definitively test which are the most effective channels for changing each of our target behaviors.

Based on the available research and the exposure theory that underpins our *Saturation+* approach, we consider spots to be the most important format to retain when scaling-up our intervention in Burkina Faso. Spots ensure that we can repeat our message throughout the day and are more cost-effective than longer formats. Multiple channels and multiple formats will always be preferable in order to achieve saturation and therefore impact, but when faced with limited resources, we suggest that spots be prioritized.

We consider short spots as the most important format to retain when scaling-up mass media campaigns.

## LIMITATIONS

We recognize there are several potential limitations to this paper. While we have described how we *intend* to adapt the intervention to broadcast at a national scale, we are not yet in a position to describe how nationwide coverage has been achieved in practice. We acknowledge that the generalizability of the described approach may vary from country to country, according to the specific media landscapes. Radio and television penetration varies widely between settings, as does the dominance of national versus local channels, and seasonal variation can also affect listening habits. Different media channels and program formats may be more suited to particular settings or to targeting particular behaviors. In certain contexts, such as those recovering from conflict, more significant time, effort, and investment in capacity building of media partners may be required, in order for radio and television stations to broadcast at the level of intensity that the *Saturation+* approach requires. Supply-side availability and quality could also affect the feasibility and impact of adopting our approach in other settings. Nonetheless, when we model the potential impact of national campaigns, we are able to adjust for and take account of variation in media penetration and service availability between different countries. Despite these limitations, the core principles that underlie the *Saturation+* approach should still be applicable to most media campaigns and across different settings.

## CONCLUSION

We have described the mass media intervention in Burkina Faso that was tested using a unique cluster RCT design, including the *Saturation+* principles and theory of change underpinning our approach, the campaign design and execution, our creative process, and the generalizable lessons learned. We have considered how best to adapt and apply this intervention at national scale. We recommend using short spot formats that can be repeated multiple times throughout the day to achieve high-intensity saturation broadcasting. We also advocate investment in sustainable energy supplies for partner community radio stations rather than paying for airtime. Whatever the efficacy outcome of the trial, for its findings to be useful to implementers and policy makers alike, it is important that we have shared these details about exactly *what* the intervention consisted of, *how* it was implemented, and what did and did not work. Much emphasis is often (rightly) placed on the importance of robust impact evaluation of public health interventions. We have addressed this by conducting the RCT in Burkina Faso, the most rigorous evaluation of a mass media campaign in a developing country. The science of delivery warrants equivalent emphasis if results are to be replicable and actionable.

## Notes

**Peer Reviewed**
